# Bibliography

**Published:** 1897-06

**Authors:** 


### Bibliography.

Artificial Anæsthesia: A Manual of Anæsthetic Agents and their Employment in the Treatment of Disease. By Laurence Turnbull, M.D., Ph.G., Aural Surgeon to the Jefferson Medical College Hospital, etc. Fourth edition, revised and enlarged. Philadelphia: P. Blakiston Son & Co., 1896.

This work practically stands alone in a very important field. It has no rival, and needs none, as it treats the subjects anæsthesia and anæsthetics most satisfactorily. There are writers who have speculated profoundly upon the chemistry and others upon the therapeutics of anæsthetics, but Dr. Turnbull, who has had much and varied clinical experience, presents his work from the standpoint of practical experience. After presenting a history of ancient and modern anæsthetics, the author gives concisely a description of all the agents that may be successfully and safely employed for inducing anæsthesia, either locally or by inhalation, and presents the chief chemical tests of the purity of each substance considered, with its composition and properties. There is also described and exhibited all the useful methods of administering the various agents, with careful and minute directions as to the precautions to be taken to avoid risk to the life of the patient and just what to do in case of danger.

The relative mortality from all the anæsthetics is also embodied, which will assist the student in forming a fair and candid opinion of this important subject.

The author very justly gives precedence to ether as the most satisfactory of all the general anæsthetics yet introduced, and, as he infers, it is somewhat remarkable that the fourth edition of this work should be issued on the fiftieth anniversary of the discovery and introduction of this agent. Full credit is given Dr. Horace Wells as the discoverer of modern anæsthesia, and the author says, "We are only now beginning to do justice to his memory."

Considerable space is given to a consideration of local anæsthetics, cocaine being treated very exhaustively, and the claims of eucaine are set forth, which, however, have not yet been thoroughly established. The work closes with a too short allusion to hypnotism and its employment as an anæsthetic agent.

This is not only a book that should be in every dental and medical library, but one that should be carefully studied by every one who assumes the responsibility of inducing insensibility.

